# The Effects of Bioactive Compounds on PTSD Treatment

**DOI:** 10.2174/011570159X333438240927103741

**Published:** 2024-10-01

**Authors:** Olha Strilbytska, Oleksandr Koliada, Volodymyr Lushchak, Oleh Lushchak

**Affiliations:** 1 Department of Biochemistry and Biotechnology, Vasyl Stefanyk Precarpathian National University, Ivano-Frankivsk, Ukraine;; 2 Department of Population Genetics, Institute of Food Biotechnology and Genomics NAS of Ukraine, Kyiv, Ukraine;; 3 Research and Development University, Ivano-Frankivsk, Ukraine

**Keywords:** PTSD, antioxidants, treatment, anxiety, depression, psychotherapy

## Abstract

Post-traumatic Stress Disorder (PTSD) is a psychiatric disease that arises in individuals who have experienced a traumatic event such as combat exposure, childhood physical abuse, sexual violence, physical assault, an accident, *etc*. Being difficult to diagnose and treat, PTSD is actively studied in areas of medicine, psychiatry, biochemistry, and rehabilitation. PTSD is characterized by significant comorbidity and is accompanied by depression and anxiety. Current treatment strategies for PTSD symptoms include psychotherapy and medications. Naturally derived compounds can offer therapeutic benefits for mood disorders without unpleasant side effects. Bioactive compounds found in food exhibit beneficial effects such as antioxidant, anti-inflammatory, and neuroprotective activities. Here, we describe the promising therapeutic benefits of a number of bioactive substances that have been evaluated in a variety of animal models and human experimental studies. Anxiolytic, antidepressant, and antidementia activities of bioactive compounds emphasize their potential for treating PTSD comorbidities. Hypothetical mechanisms of actions are also discussed, providing insights into their potential for human mental health.

## 1 INTRODUCTION

Traumatic, shocking, or dangerous events can trigger post-traumatic stress disorder (PTSD), which is an actively studied field in medicine, psychiatry, biochemistry, and rehabilitation [1]. The Diagnostic and Statistical Manual of Mental Disorders (DSM-5) describes PTSD as an anxiety disorder included in a new class of “Trauma- and Stressor-related Disorders.” According to the DSM-5, specific criteria are required for the diagnosis of PTSD, including “A” – trauma criterion, “B” – intrusive recollection criterion, “C” – avoidance criterion, “D” – negative cognitions and mood criterion, “E” – alterations in arousal or reactivity criterion [2]. Individuals with PTSD exhibit clinically relevant alexithymia, suicide ideation, and affective temperaments [3]. Higher PTSD prevalence is confirmed among refugees, persons suffering from mental disorders, and socioeconomically unprotected populations [4]. A recent nationwide cross-sectional study of the prevalence of stress, anxiety, and symptoms of PTSD among Ukrainians after the first year of the Russian invasion showed that 93% of surveyed people had faced at least one of the measured mental health issues at moderate or severe levels [5].

PTSD is recognized for its significant comorbidities. Mental health problems accompanying PTSD include depression and anxiety [6]. The main symptoms of PTSD are flashbacks, avoidance, intrusive thoughts, nightmares, impaired concentration, irritability, aggression, negative thoughts, and loss of interest in life. Most of these symptoms are common to anxiety, depression, and PTSD, which make it difficult to diagnose. However, at present, PTSD is diagnosed only by subjective psychological parameters, which can lead to misdiagnosis. Therefore, there is an urgent need to develop objective laboratory methods that will help detect acute PTSD based on changes in biochemical and immunological parameters.

Despite the large number of pharmacological and non-pharmacological approaches that have been used to treat PTSD patients so far, there is no effective treatment that can be extensively used. Antioxidant, anti-inflammatory, anti-tumor, and cardioprotective activities have been reported for various phytoactive compounds, including resveratrol, quercetin, curcumin, catechin, *etc*. [7-9]. Here, we discuss alternative pharmacological methods of treatment using bioactive compounds that exist in foods and varied medicinal and non-medicinal plants. Reduction of oxidative stress, inflammation as well as beneficial metabolic alteration are considered as the main molecular mechanisms mediating the therapeutic effects of bioactive compounds for PTSD treatment.

## 2 MATERIALS AND METHODS

The data for the review were collected using academic search systems PubMed and Google Scholar. The search terms used were: “Post-Traumatic Stress Disorder,” “Central Nervous System,” “Bioactive Compounds,” “Depression,” “Anxiety,” “PTSD treatment,” “PTSD symptoms,” and “Psychotherapy”. The initial screening was based on article titles and abstracts, followed by a full-text review of the literature. Only bioactive compounds that showed clear-cut behavioral effects in animal models and/or in human tests were included in this review. Twenty-six bioactive compounds were selected and described as having anxiolytic and antidepressant activities. Bioactive compounds, dose range, animals, models, beneficial health effects, possible mechanisms of action, and references are shown in Table **1**.

Most of the papers discussed here are pertinent to bioactive compound research. All papers were published in peer-reviewed journals, with approximately 85% published from 2014 to 2024.

## 3 ETIOLOGY, SYMPTOMS, AND COMORBIDITY OF PTSD

A vast number of factors contribute to PTSD development. Female gender, childhood trauma, low socioeconomic status, and poor education are the main risk factors for PTSD development and progression [10]. PTSD combines psychological, physiological, and biochemical components, while the molecular mechanisms of PTSD and its long-term consequences for public health are poorly understood. Recent studies have discussed that PTSD is accompanied by metabolic disorders, decreased immune system efficiency, cardiovascular dysfunction, *etc.* [11]. PTSD also leads to premature aging and is associated with a higher risk of age-related diseases. Morphological and functional changes in specific brain regions, including the prefrontal cortex, amygdala, and hippocampus, play a certain role in the development of PTSD symptoms [12]. Structural perturbations in brain regions that are involved in stress response were demonstrated. Indeed, reduced gray matter volume of the hippocampus, amygdala, and anterior cingulate cortex was shown in persons with diagnosed PTSD [13].

Changes within the regulatory triangle of nervous, endocrine, and immune systems targeting relevant pathophysiological mechanisms of PTSD [14]. The hypothalamus-pituitary-adrenal (HPA) axis and the sympathetic nervous system (SNS) are the main physiological pathways that are involved in the regulation of mood disorders [15]. Stress causes an increase in the secretion of stress hormones, including adrenaline and epinephrine, in the circulation, causing brain changes and triggering stress response. Corticotropin-releasing hormone (CRH) is secreted by the neurons in the paraventricular nucleus of the hypothalamus [16]. CRH stimulates the secretion of adrenocorticotropic hormone (ACTH) from the anterior pituitary gland. The circulation brings ACTH to the adrenal glands and induces cortisol release [17], which modulates metabolic and immune processes and provides organismal stress response [18]. However, PTSD is associated with lower cortisol levels [19]. Cortisol regulates its own production through negative feedback inhibiting the sensitivity of glucocorticoid receptors [20]. Cortisol level is not an appropriate PTSD marker but significantly contributes to PTSD symptomatics. Several symptoms depend on cortisol levels, including intrusion, avoidance, and impairment in cognition and mood [21].

## 4 PTSD TREATMENT STRATEGIES

PTSD diagnosis is based on specific criteria within DSM-5 that describe specific symptoms that persist for more than one month. Screening of PTSD includes examination of mental status through impaired sleep and concentration, nightmares, flashbacks related to the event, negative emotions avoidance, and increased awareness. The magnitude of the mental health problem requires a much broader understanding of the causes, mechanisms, and progression of stress disorders. Psychotherapy, obviously, plays the main role in the treatment of PTSD and other stress-related disorders, but additional pharmacological interventions can significantly enhance its effectiveness or even reduce the manifestations of the disorder on their own when psychotherapy cannot be provided.

The first-line treatment of PTSD symptoms involves trauma-focused psychotherapy. Psychological therapies include exposure therapies, anxiety management training (AMT), eye movement desensitization and reprocessing (EMDR), trauma-focused cognitive-behavioral therapy (CBT), cognitive processing therapy (CPT), and imaginal exposure [10]. Exposure therapy involves returning to the place of the traumatic event to minimize avoidance and promote mastery [22]. AMT is an effective, short-term behavioral program based on self-control therapy. Techniques within the AMT program help people manage their anxiety; this includes belly breathing techniques, guided self-dialogue, communication skills training, and others [22]. Self-soothing skills are developed using anger management training within the AMT program [23].

EMDR therapy is a mental health treatment technique that involves moving your eyes a specific way while processing traumatic memories [24]. Eyes are moving horizontally or vertically back and forth in combination with thinking about a trauma. EMDR helps the brain to process unpleasant incidents. The procedure is applied until shifts occur in the way that person experiences that memory, and more information from the past is processed.

Trauma-focused CBT is often used to reduce PTSD symptoms among children and adolescents. The trauma-focused CBT technique is based on two critical developmental aspects: the role of the caregiver and the nature of regulation of the emotion [25]. Children and caregivers learn new skills to process these traumatic events, control unwanted feelings, and enhance safety and communication. Core components of Trauma-focused CBT include psychoeducation and parenting skills, relaxation, cognitive processing of the trauma, trauma narrative, conjoint child-parent sessions, and enhancing future safety and development. This treatment is short-term and lasts no more than 16 sessions. Indeed, more than 80% of traumatized children feel improvement after 12-16 weeks of treatment [26].

CPT is a specific type of CBT that is an effective strategy in reducing symptoms of PTSD. CPT is a highly structured treatment approach and consists of 12 weekly sessions. These CPT sessions can take place in a group, one-on-one, or in a combined group. Treatment begins with psychoeducation about trauma, thoughts, and emotions [27]. Next, patients are taught the connection between events, thoughts, and feelings, including all sensory details. The final stage includes the development of skills to identify and address unhelpful thinking, focusing on safety, trust, power, control, esteem, and intimacy. CPT procedures create a new understanding and conceptualization of the traumatic event that reduces negative effects on current life.

The imaginal exposure strategy is realized *via* repeated and prolonged recounts of the story of the trauma memory during the session of 30-45 minutes [28]. During sessions, people retell the full story of the trauma in the present tense several times in detail, including thoughts. The repetitions lead to the reduction of the fear response through the attenuation processes [29].

Psychotherapy is not always effective in the treatment of PTSD, and discovering role of pharmacotherapy has been studied more extensively in recent years. Medication can be applied in conjunction with psychotherapy to enhance effectiveness. Selective serotonin reuptake inhibitors (SSRI) and serotonin-norepinephrine reuptake inhibitors (SNRI) are classes of first-line medications used to alleviate symptoms of depression, anxiety, and PTSD [10, 30].

Serotonin and serotonergic systems regulate various functions of the brain and are involved in the regulation of various physiological processes, including mood, cognition, anxiety, learning, memory, and sleep. Serotonin deficit contributes to the pathophysiology of mood disorders, including PTSD. SSRIs enhance serotonergic activity and increase serotonin levels in the peripheral and central nervous systems (CNS) by preventing its uptake by the presynaptic neurons [31]. Serotonin is available to bind to postsynaptic neurons and benefit mood. SSRIs improve stress resilience, decreasing sensitivity to stressors [32]. Sertraline, paroxetine, and fluoxetine are the most effective SSRIs. However, only sertraline (Zoloft) and paroxetine (Paxil) are approved by the Food and Drug Administration for PTSD treatment [33].

Fluoxetine, which belongs to the SNRI class, is not approved for the treatment of PTSD but is often used as off-label PTSD therapy [34]. At lower doses, fluoxetine inhibits serotonin reuptake; conversely, at higher doses, it inhibits norepinephrine reuptake, and in this way, fluoxetine contributes to the balance of serotonergic and noradrenergic neurotransmission [31].

Second-line treatments are mirtazapine, nefazodone, tricyclic antidepressants (TCAs), or monoamine oxidase (MAO) inhibitors. TCAs were shown to improve PTSD symptoms by maintaining a balance of the neurotransmitters. Indeed, TCAs inhibit the reabsorption of serotonin and norepinephrine [35]. TCAs can cause more significant adverse effects as compared to first-line medications due to their anticholinergic activity. Amitriptyline and imipramine as TCAs have received FDA approval for the treatment of MDD [35].

MAO inhibitors have antidepressant activity due to inhibition of the action of MAO enzymes that lead to increased serotonin, norepinephrine, and dopamine concentrations [36]. Phenelzine is effective in alleviating PTSD intrusive symptoms, including intrusive dreams or memories of a traumatic event [37]. However, MAO inhibitors have serious side effects and, in combination with SSRIs, cause serotonin syndrome, which is potentially lethal [38].

The medications used to treat PTSD can cause side effects that depend on the type of medication. However, side effects from SSRIs and SNRIs are similar and include dry mouth, dizziness, headache, fatigue, difficulty sleeping, changes in sexual function, weight changes, *etc*. Рotential benefit of the medication must be considered with the potential side effects and difference in therapeutic response of individual patients.

## 5 USE OF BIOACTIVE COMPOUNDS FOR PTSD THERAPY

**Fig. (1):**
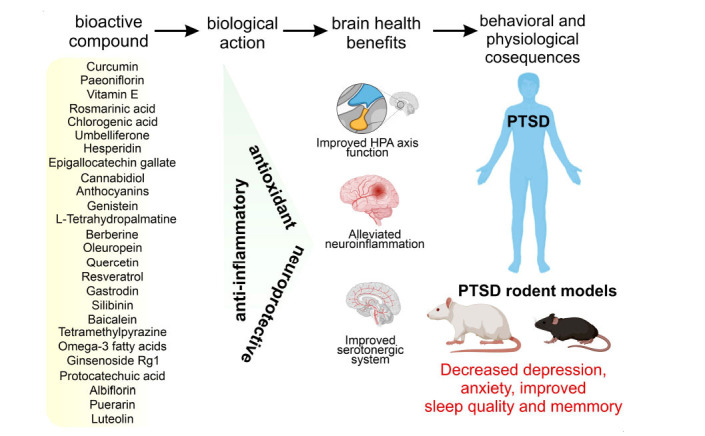
Bioactive compounds and their influence on PTSD. Bioactive compounds have antioxidant, anti-inflammatory, and neuroprotective effects that are manifested in brain health benefits. Enhanced function of HPA axis, serotonergic system, and alleviated neuroinflammation result in decreased anxiety, depression and improved cognition and sleep quality.

Nutrition is one of the main factors that affect health. Herbal medicines were shown to have significant neuroprotective, anxiolytic, and anti-depressant effects in animal models of PTSD. However, plant extracts contain a wide variety of bioactive substances and their pharmacological activities [7]. Here, we focus on specific categories of bioactive compounds and their pharmacological activities (Fig. **1**). Bioactive compounds show significant efficiency in reducing PTSD symptoms as compared to approved medications but with fewer pronounced side effects. The therapeutic benefits of a number of bioactive substances on PTSD symptoms are summarized in Table **1**.

Cannabidiol (CBD), also known as “medical marijuana”, exerts anti-inflammatory, antioxidant, and neuroprotective properties [39]. CBD originates from cannabis plants, including *Cannabis sativa*. Preclinical studies suggested that CBD may help reduce and manage symptoms of PTSD in animal models. Anxiety and anxiety-induced sleep disturbances caused by repeated combination tests (RCT) in rats were alleviated by CBD treatment [40]. Nasal administration of complex CBD and temperature-sensitive hydrogels (30 mg/kg) significantly decreases anxiety in mice exposed to foot shock [41]. Several human studies are devoted to the effects of CBD on PTSD symptoms [42-44]. Oral CBD administration of 11 adult patients with PTSD in conjunction with psychotherapy leads to a decrease in PTSD symptom severity [42]. The study by Shannon and Oplia-Lehman [43] showed a reduction in anxiety symptoms and sleep disturbance in 10-year-old patients with PTSD due to sexual trauma after oral administration of 12-37 mg CBD daily. A clinical trial of 150 participants with PTSD showed that 300 mg of CBD is beneficial in the treatment of PTSD [44]. The beneficial effects of CBD on PTSD symptoms may involve serotoninergic transmission *via* 5-HT receptors [45]. Moreover, stimulation of the adenosinergic system by CBD treatment results in suppression of aversive memory expression [46]. Reduction in the hyperactivity of the amygdala and mPFC is involved in mechanisms of CBD action against PTSD symptoms [47].

Oleuropein is the main phenolic component of olive leaves (*Olea europaea*). Antioxidant, anti-inflammatory, and anti-diabetic properties were shown for oleuropein [48]. Moreover, oleuropein exhibits neuropsychiatric activities, improving anxiety-like symptoms following single prolonged stress (SPS) exposure in rats [49]. In the study of Lee and colleagues [49], SPS-exposed male rats were treated with oleuropein daily for 14 days, which resulted in a reduced anxiety index, decreased grooming behavior, and reduced anxiety-like behavior in the elevated plus maze (EPM) test [49]. The anxiolytic actions of oleuropein may be associated with the restored level of serotonin and neuropeptide Y in rat hippocampus, which was depleted after SPS [49].

Anthocyanins that are present in berries were shown to have a beneficial effect on mental health problems *via* an inhibitory effect on monoamine oxidase enzymes [50]. Daily consumption of anthocyanin-rich mulberry milk alleviates various mood and anxiety symptoms in healthy participants aged 18-60 years [50]. Anthocyanin from purple cauliflower was effective in decreasing depression symptoms in mice [51]. Female mice were exposed to chronic unpredictable mild stress (CUMS) for after 3 weeks and treated with anthocyanin from purple cauliflower with a total anthocyanin content of 50, 100, or 200 mg/kg [51]. Inhibition of depression-like behaviors following anthocyanin treatment was shown using sucrose preference test, forced swimming test, and EPM test [51]. Inhibition of MAO enzymes and promotion of neurogenesis and dendrite development in the hippocampus is the main mechanism of the beneficial effects of anthocyanin on alleviating PTSD symptoms [51].

Curcumin, a major compound found in *Curcuma longa*, has numerous therapeutic properties, including antioxidative, anti-diabetic, and anti-inflammatory activities, and modulates multiple cell signaling pathways [52, 53]. Using the PTSD rat model, it was established the anxiolytic-like effects of curcumin [54]. Rats were subjected to SPS with subsequent curcumin treatment at doses of 20, 50, or 100 mg/kg once daily for 14 days [54]. Decreased grooming behavior, reduced anxiety index, and increased number of open-arm visits on the EPM test were found after curcumin treatment of PTSD-rats [54]. The serotonergic receptors that are implicated in the pathophysiology of PTSD [55] were activated in the hippocampus and amygdala following curcumin treatment [54].

Genistein is an isoflavone that is found in beans, soy, and coffee [56] and has antioxidant, anti-inflammatory, and neuroprotective properties. The study of Lee and colleagues [57] suggested genistein as an effective therapeutic biomolecule for the treatment of PTSD. Male rats were subjected to SPS with subsequent genistein administration at doses 2, 4, and 10 mg/kg for 14 days [57]. Using Morris water maze (MWM) and object recognition task (ORT) tests, it was found to significantly improve cognitive function in rats after genistein treatment [57]. Improvement of spatial memory and learning disabilities caused by PTSD in genistein-treated rats was associated with enhanced serotonin levels in the medial prefrontal cortex and hippocampus [57]. Anti-anxiety activity was found in the study of Wu and colleagues [58]. Electric foot shock was used to induce PTSD symptoms, and genistein was injected intraperitoneally daily for 7 days at a range of doses of 2-8 mg/kg [58]. Genistein attenuates natural anxiety in the EPM test, enhances serotonin secretion, and improves the serotonergic transmission system in the amygdala [58].

Berberine is one of the main alkaloids extracted from several plants and is known for neuroprotective, antioxidant, anti-inflammatory, and antidiabetic activities. The study by Lee and colleagues [59] demonstrated the anxiolytic effects of berberine in rats with PTSD symptoms. PTSD phenotype in rats was induced by exposure to SPS, and berberine was received intraperitoneally at doses of 10, 20, or 30 mg/kg once daily for 14 days [59]. Reduced anxiety in PTSD rats under berberine treatment is associated with enhancing dopamine expression in the hippocampus and striatum [59].

Tetrahydropalmatine is an alkaloid primarily found in the plants of the *Corydalis* genus and is famous for antidepressant, anxiolytic, cardioprotective, and neuromodulatory activities. Tetrahydropalmatine increased time spent in open arms of EPM decreased grooming behavior, and immobility in PTSD rat model [60]. In the study of Lee and colleagues [60], anxiety and depression were induced by SPS, and tetrahydropalmatine was administered intraperitoneally at doses 10, 20, and 50 mg/kg body weight. The mechanisms of anxiolytic and antidepressant activity of tetrahydropalmatine involve restriction of the decrease in neuropeptide Y level and the increase in corticotrophin-releasing factor expression in the hypothalamus [60]. The restraint/shock stress model of PTSD demonstrated a significant decrease in anxiety and changes in rat brain gene expression under tetrahydropalmatine treatment [61, 62]. Tetramethylpyrazine is a vital alkaloid in traditional Chinese medicine and showed beneficial effects on symptoms of anxiety in rats after SPS exposure [63]. Anti-PTSD effects of tetramethylpyrazine (40 mg/kg) were associated with reversing the serotonin level and restoring HPA axis function [63].

Using various animal models, the anxiolytic and antidepressant effects of quercetin were demonstrated. Activation of corticotrophin-releasing factor (CRF) release from the hypothalamus causes anxiogenic- and depressant-like effects in mice [64]. The study by Kumar and Goyal [65] reported that quercetin attenuates stress-induced behavioral depression and anxiety through the modulation of CRF. Neurotransmitter systems like GABA, nitric oxide, and serotonin are involved in quercetin-induced beneficial behavioral effects [66]. EPM test showed the anxiolytic effect of quercetin in pregnant female rats previously exposed to a predator, which was associated with a significant decrease in corticosterone level and alleviation of oxidative stress [67].

The beneficial effects of omega-3 fatty acids (OFA) on the structure and function of the brain were shown [68]. OFA improves mental health performance *via* the increase in the production of neurotransmitters [69]. The PTSD symptoms were induced in rats using an SPS, and OFA was administered orally at a dose of 100 mg/100 g body weight/day. The study showed that OFA prevented memory impairment induced by SPS in rats, which is associated with antioxidant mechanisms in the hippocampus [69].

Resveratrol is a natural polyphenol with a wide range of biological activities, including antioxidant, anti-inflammatory, and neuroprotective effects [70, 71]. It is worth noting that both resveratrol and SSRIs affect serotonin transporter with equal intensity, correcting anxiety symptoms within PTSD [72]. Using rats exposed to chronic predator stress, the protective effect of resveratrol against PTSD was shown to be accompanied by higher production of neurotrophins in the brain and decreased activity of 11-β-hydroxysteroid dehydrogenase type 1 in the liver [72]. Resveratrol improves HPA axis functioning and activates the expression of downstream neuroprotective molecules, including cAMP response element binding protein (pCREB) and brain-derived neurotrophic factor (BDNF) [73]. The electric foot shock mice model of PTSD was used to show resveratrol's effectiveness on depression and anxiety [74]. Resveratrol at doses 20 and 40 mg/kg decreased anxiety and fear memory in an animal PTSD model that was associated with decreased HPA axis stress hormone levels, including corticosterone, corticotropin-releasing hormone, and adrenocorticotropic hormone [74].

Ginsenoside Rg1 (GRg1) is one of the primary bioactive components in ginseng, and it has neuroprotective, anti-aging, anti-inflammation, and antioxidative effects [75]. A set of behavioral tests demonstrated that GRg1 administration promoted fear extinction and alleviated depression symptoms in SPS mice [76]. Recovery of hippocampal synaptic plasticity, reduction of Kir4.1 level, and pro-inflammatory cytokines are the mechanisms related to GRg1 potential in the treatment of mental disorders [76]. Oral administration by GRg1 at a dose of 5 mg/kg was effective in preventing PTSD-like behavior induced by electric shock [77]. Moreover, GRg1 suppresses the levels of corticotropic hormone and corticosteroids, suggesting the HPA axis is the involved mechanism [76]. The anxiolytic action of GRg1 was confirmed using the SPS rat model [78].

Gastrodin is the bioactive component isolated from rhizomes of the *Gastrodia elata* and has an anti-oxidant and anti-inflammatory action. High doses of gastrodin (200 mg/kg daily) ameliorated SPS-induced PTSD-like behavior in rats [79]. Prevention of anxiety-like behavior by gastrodin was associated with with the decrease of IL-1β and IL-6 levels in the hippocampus [79]. Gastrodin improved learning and spatial memory impairment and decreased anxiety and fear in rat models of PTSD [80]. Overexpression of autophagy-related genes Beclin-1 and LC3, as well as depressed expressions of Bcl-2 and p62 mPFC neurons, suggested the involvement of autophagy in mPFC neurons in beneficial effects of gastrodin on PTSD manifestation [80].

Hesperidin is a bioflavonoid compound isolated from citrus fruits with numerous biological properties, such as anti-inflammatory and antioxidant effects. Using the open field test and forced swimming test it was found that daily hesperidin administration significantly improved depression-like behaviors in the PTSD rat model [81]. A significant increase in serotonin levels in the hippocampus in rats treated with hesperidin after exposure to SPS was observed in the study of Lee and colleagues [81]. Antidepressant-like effects of hesperidin were associated with a decrease in interleukin-1β (IL-1β)/IL-6/TNF-α levels and higher levels of hippocampal brain-derived neurotrophic factor (BDNF) [82]. Decreasing cerebral oxidative stress [83], hesperidin suppresses anxiety in the PTSD rat model.

Silibinin, a flavonoid isolated from the *Silybum marianum,* is considered to be a useful therapeutic compound for the treatment of anxiety and depression caused by PTSD [84]. SPS-induced PTSD symptoms are ameliorated by silibinin exposure by increasing serotonin levels [84].

Umbelliferone is a natural coumarin-derived compound with a wide range of biological actions. Using open field test and the forced swimming test, Lee and colleagues [85] showed that daily umbelliferone administration at a dose of 60 mg/kg improved depression-like behaviors and enhanced serotonin levels in the hippocampus and amygdala. Alleviation of depressive-like behaviors under umbelliferone treatment (15 mg/kg, 30 mg/kg) was also found in rats exposed to CUMS [86]. The mechanisms of neuroprotective effects on CUMS-induced model of depression involve the reduction of neuronal apoptosis and inflammatory cytokines levels [86].

Baicalein is a flavonoid found in Chinese herbs, such as Scutellaria baicalensis, and exhibits a variety of biological effects, including anti-inflammatory and antioxidant [87]. The anxiolytic effect of baicalein was found using a mouse model of PTSD [88]. A set of behavior tests showed that baicalein treatment SPS induced PTSD-like behaviors in mice [88]. The anxiolytic effect of baicalein is associated with increased noradrenaline and serotonin levels [88].

Chlorogenic acid is a natural compound from higher plants with potent antioxidant and anti-inflammation properties [89]. A clinical study showed a positive effect of chlorogenic acid on mood and attention processes in persons with PTSD symptoms [90]. Chlorogenic acid at a dose of 20 mg/kg decreases anxiety-related behaviors in male mice [91]. The study of Bouayed and colleagues [91] also showed that chlorogenic acid protected granulocytes from oxidative stress. Chlorogenic acid attenuates depression-like behavior in ACTH-induced depression rats [92]. Improvement of learning, memory, and cognitive impairment under chlorogenic acid was shown to be realized *via* anti-acetylcholinesterase and anti-oxidative activities in mice [93].

Luteolin, naturally found in a variety of foods, especially oranges, is a flavonoid that exerts antioxidant and anti-inflammatory properties. Luteolin decreases fear, depression, and anxiety in SPS-induced rats of the PTSD model [94]. Administration of luteolin leads to suppression of SPS-induced increase in plasma corticosterone and adrenocorticotropic hormone levels [94]. Regulation of the HPA axis and monoamine balance is the potential mechanism of the anti-PTSD-like effect of luteolin [94].

Albiflorin is a major monoterpene glycoside isolated from *Paeonia lactiflora* and is effective in the therapy of depression [95]. SPS-induced PTSD-like behavior in rats can be alleviated by albiflorin (7.0 and 14 mg/kg) [95]. The anti-PTSD-like effect of albiflorin is associated with an increase in the levels of allopregnanolone in the prefrontal cortex, hippocampus, and amygdala [95].

Antioxidant, anti-inflammatory, and analgesic action were shown for paeoniflorin, which is the major active component extracted from *Paeonia lactiflora* [96]. PTSD-like behavioral anxiety was ameliorated in SPS-exposed rats by paeoniflorin treatment (10 mg/kg, 20 mg/kg) [96]. SPS-induced higher levels of corticosterone, corticotropin-releasing hormone, and adrenocorticotropic hormone were blocked by paeoniflorin [97]. HPA axis and serotonergic system activation were proposed as the main mechanisms of the anxiolytic action of paeoniflorin [97].

Rosmarinic acid is a polyphenol usually found in herbal plants and is famous for its anti-inflammatory and antioxidant activities [98]. Therapeutic effects of rosmarinic acid in mood disorders were found in the PTSD rat model [99]. Enhanced SPS-induced PTSD symptoms were alleviated by rosmarinic acid (10 mg/kg) treatment [98]. Moreover, rosmarinic acid promotes cell proliferation in the hippocampus and increases pERK1/2 expression [99].

The study by Sur and colleagues [100] evaluated the efficacy of protocatechuic acid using rat models of PTSD. Protocatechuic acid is a major metabolite of antioxidant polyphenols found in green tea with a wide range of biological activities. Fear extinction, antidepressant, and anxiolytic effects were found in rats exposed to SPS with subsequent protocatechuic acid administration (100 or 200 mg/kg) [100]. Protocatechuic acid maintains the balance of serotonin and norepinephrine in the medial prefrontal cortex and hippocampus and blocks the decrease in expression of BDNF in the hippocampus [100].

Antidepressant- and anxiolytic-like effects of puerarin have been reported in the study of Su and colleagues [101]. Puerarin, a natural compound obtained from the root of *Pueraria lobate,* reduces anxiety and contextual fear in rats exposed to SPS at doses 50 and 100 mg/kg [101]. The mechanisms of anti-PTSD effects of puerarin involve the modulation of neurosteroid biosynthesis and stress hormones in the HPA axis [101].

Vitamin E is a lipid-soluble vitamin presented in food components and is known for its powerful antioxidant activity. Considering the antioxidant and anti-inflammatory role of vitamin E, it is very effective in the prevention and alleviation of various disease symptoms [102]. PTSD, which is associated with oxidative stress and neuroinflammation [1], could be prevented at behavioral and molecular levels through vitamin A treatment. The SPS animal model of PTSD showed the beneficial effects of oral vitamin E administration at a dose of 100 mg/kg on spatial memory impairments [103].

The most abundant polyphenol in green tea is *epigallocatechin gallate*, which has a strong antioxidant action. Improved learning and memory deficit in the SPS rat model of PTSD was found after epigallocatechin gallate administration (25 mg/kg) [104]. Improved HPA axis functioning and decreased neuroinflammation are involved in the beneficial effects of epigallocatechin gallate in the treatment of PTSD symptoms [104].

## 6 FUTURE RESEARCH DIRECTIONS

The early development processes that regulate the susceptibility to depression and anxiety will better clarify the pathophysiological processes that regulate the susceptibility to PTSD. Recent work highlighted the role of alterations in the hypothalamic paraventricular nucleus (PVN) as an important determinant of how stress interfaces with emotional dysregulation [105]. The PVN is an important site for the initiation of the HPA axis and its interactions with PVN oxytocin neurons. Recent work shows oxytocin levels to be decreased in PTSD [106], with intranasal oxytocin having utility in PTSD treatment [107].

PTSD is frequently conceptualized as accelerating aging [108], with recent work showing aging to be intimately linked to alterations in the night-time processes of dampening and resetting body systems for the upcoming day [109]. This may be mediated by variations in the interface of melatonin, gut microbiome-derived butyrate and bcl2-associated athanogene (BAG)1 with the wider cortisol system [110]. The clarification of such putative underpinnings to alterations in the affective state in the etiology and ongoing pathophysiology of PTSD should better clarify the effects of the natural products reviewed above, as well as provide novel pharmaceutical and nutriceutical targets, as well as integrate the benefits of current treatments, such as the kappa-opioid receptor antagonist, buprenorphine [111].

More clinical trials are needed to confirm the functional properties of bioactive compounds in individuals with PTSD. Understanding the pharmacological effects and mechanisms of action of bioactive compounds can contribute to the development of novel drugs and functional food supplements. Further research should explore the bioavailability of bioactive compounds in pharmaceutical companies. Future research regarding bioactive compounds for PTSD treatment should also focus on experiments with more variable groups in terms of age, health status, gender, or weight. The ultimate goal is to develop sustainable and healthier products that can be used for the treatment of mental health conditions.

## CONCLUSION AND PERSPECTIVES

Recent experimental studies indicate about growing popularity of bioactive compounds for a variety of chronic diseases. Naturally derived components show benefits with fewer side effects as compared to the FDA-approved pharmacological therapy of mental health complications. However, every naturally derived component should be carefully tested and scientifically approved before being widely used. Each bioactive component should be carefully tested for dosage, possible side effects, and contraindications. Many bioactive compounds act in a concentration-dependent manner. Even very useful drugs might be effective in specific dose ranges. The doses below the lower limit in this range are ineffective, whereas doses higher than the upper limit cause adverse effects. Biologically active supplements do not always have beneficial properties that may depend on specific nutritional regimens. For this reason, it is extremely important to select the optimal set of biologically active ingredients that would be accompanied by a directly defined dietary formulation. Additionally, the individual’s physical health status should be carefully considered before using any bioactive compound. To minimize health risks, none of these substances should be used during pregnancy or breastfeeding.

Dietary components modulating the inflammation, oxidative stress, and metabolic complications under PTSD would slow down the disease progression. Consequently, biologically active compounds may be considered as a candidate pharmacological adjunct to psychological therapies for PTSD. Future studies are needed to evaluate bioactive compounds in clinical studies.

## Figures and Tables

**Table 1:** Bioactive compounds used to decrease PTSD symptoms.

**Bioactive Components**	**Mechanism of Action**	**PTSD Symptoms**	**Model/Human**	**References**
Cannabidiol	Improving serotonergic and adenosinergic systems	Decreased complex PTSD symptoms	11 patients with PTSD	Elms *et al*., (2019)
			150 patients with PTSD	Telch *et al*., (2022)
		Decreased anxiety and insomnia	10-year-old girls with PTSD caused by sexual abuse	Shannon and Oplia-Lehman, (2016)
		Decreased anxiety and insomnia	RCT Rats	Hsiao *et al*., (2012)
		Decreased anxiety	Foot shock Mice	Pang *et al*., (2021)
Oleuropein	Maintaining the level of serotonin and neuropeptide Y level in the hippocampus	Decreased anxiety	SPS Rats	Lee *et al*., (2018a)
Anthocyanin	Inhibition of MAO enzymes	Decreased anxiety and depression	Healthy participants	Rangseekajee *et al*., (2024)
		Depression	CUMS-exposed mice	Fang *et al*., (2020)
Curcumin	Activation of serotonin receptors in the hippocampus and amygdala	Decreased anxiety	SPS Rats	Lee and Lee, (2018)
Genistein	Maintaining serotonin level	Improved cognition	SPS Rats	Lee *et al*., (2020)
	Improvement serotonin secretion and enhancing serotonergic system	Decreased anxiety	Foot shock Rats	Wu *et al*., (2017)
Berberine	Enhancing dopamine expression	Decreased anxiety	SPS Rats	Lee *et al*., (2018b)
L-Tetrahydropalmatine	Activation of the HPA axis	Decreased anxiety and depression	SPS Rats	Lee *et al*., (2014)
	Changes in rat brain gene expression	Decreased anxiety	Restraint/shock Rats	Ceremuga *et al*., (2014)
Tetramethylpyrazine	Maintaining serotonin levels and restoring HPA axis function	Decreased anxiety	SPS Rats	Lee *et al*., (2018)
Quercetin	GABA, nitric oxide, and serotonin systems	Decreased anxiety and depression	CRF-induced anxiety and depression mice	Bhutada *et al*., (2010)
			Immobilization mice	Kumar and Goyal, (2008)
	Maintaining corticosterone levels, alleviation of oxidative stress	Decreased anxiety	Pregnant female rats acutely stressed by a predator	Ma *et al*., (2021)
Omega-3 fatty acids	Antioxidant mechanisms in the hippocampus	Improved memory	SPS Rats	Alquraan *et al*., (2019)
Resveratrol	Enhancing serotonergic system	Decreased anxiety	Chronic predator stress rats	Tseilikman *et al*., (2023)
	Prevention of hypothalamic-pituitary-adrenal axis dysfunction	Decreased anxiety and depression	Electric foot shock Mice	Zhang *et al*., 2017)
Ginsenoside Rg1	Improving hippocampal synaptic plasticity, reduction of Kir4.1 level, and anti-inflammatory action	Prevented depression	SPS Mice	Zhang *et al*., (2021)
	Decreasing corticosterone and corticotrophin-releasing hormone levels	Decreased complex PTSD symptoms	Electric foot shock Mice	Wang *et al*., (2015)
	Improving the HPA axis function	Decreased anxiety	SPS Rats	Lee *et al*., (2016)
Gastrodin	Reduction of hippocampal inflammation	Decreased anxiety	SPS Rats	Peng *et al*., (2013)
	Autophagy in mPFC neurons	Decreased anxiety; improved learning and spatial memory	SPS Rats	Lei *et al*., (2020)
Hesperidin	Enhancing serotonergic system	Decreased anxiety and depression, improved memory	SPS Rats	Lee *et al*., (2021)
Silibinin	Enhancing levels of serotonin in the hippocampus	Decreased anxiety and depression	SPS Rats	Lee *et al*., (2020)
Umbelliferone	Enhancing serotonin levels in the hippocampus and amygdala	Decreased depression	SPS Rats	Lee *et al*., (2020)
	Reduction of neuronal apoptosis and inhibition of inflammatory cytokines	Decreased depression	CUMS Rats	Qin *et al*., (2016)
Baicalein	Enhancing the noradrenaline and serotonin levels	Decreased anxiety	SPS Mice	Ruan *et al*., (2023)
Chlorogenic acid	Antioxidant action	Decreased anxiety	Mice	Bouayed *et al*., (2007)
Luteolin	-	Reduced fear, anxiety, and depression	SPS Rats	Sur and Lee, (2022)
Albiflorin	Improving the HPA axis function	Decreased complex PTSD symptoms	SPS Rats	Qiu *et al*., (2017)
Paeoniflorin	Activation of the HPA axis and serotonergic system	Decreased anxiety	SPS Rats	Qiu *et al*., (2018)
Rosmarinic acid	Increased hippocampal cell proliferation	Decreased anxiety and complex PTSD symptoms	SPS Rats	Nie *et al*., (2014)
Protocatechuic acid	Enhancing serotonergic system	Reduced fear, anxiety, and depression	SPS Rats	Sur *et al*., (2022)
Puerarin	Improving the HPA axis function	Reduced complex PTSD symptoms	SPS Rats	Su *et al*., (2019)
Vitamin E	Antioxidant action	Prevented memory impairment	SPS Rats	Ahmed *et al*., (2020)
Epigallocatechin gallate	Preventing neuroinflammation; improving HPA axis function	Stimulated learning and memory deficit	SPS Rats	Lee *et al*., (2018)

## LIST OF ABBREVIATIONS

ACTH: Adrenocorticotropic Hormone
AMT: Anxiety Management Training
BDNF: Brain-derived Neurotrophic Factor
CBT: Cognitive-behavioral Therapy
CNS: Central Nervous Systems
CRH: Corticotropin-releasing Hormone
EPM: Elevated Plus Maze
HPA: Hypothalamus-pituitary-adrenal
MAO: Monoamine Oxidase
PTSD: Post-traumatic Stress Disorder
RCT: Repeated Combination Tests
SNS: Sympathetic Nervous System
SSRI: Selective Serotonin Reuptake Inhibitors
TCAs: Tricyclic Antidepressants
